# COVID-19: more than a cytokine storm

**DOI:** 10.1186/s13054-020-03267-w

**Published:** 2020-09-04

**Authors:** Giovanni Riva, Vincenzo Nasillo, Enrico Tagliafico, Tommaso Trenti, Patrizia Comoli, Mario Luppi

**Affiliations:** 1 Department of Laboratory Medicine and Pathology, AUSL/AOU Policlinico, Modena, Italy; 2 Department of Medical and Surgical Sciences, University of Modena and Reggio Emilia; Hematology Unit, AOU Policlinico, Via Del Pozzo 71, 41124 Modena, Italy; 3 Pediatric Hematology/Oncology Unit, IRCCS Policlinico San Matteo, Pavia, Italy

## Background

In these first months of coronavirus disease-19 (COVID-19) pandemic, a mainstream pathogenetic hypothesis, likely stemming from early clinico-therapeutic observations, has been suggesting that severe COVID-19 may represent a sort of hyperimmune disorder, akin, in particular, to secondary hemophagocytic lymphohistiocytosis (sHLH) and macrophage activation syndrome (MAS) [1–3]. In this view, COVID-19-associated cytokine storm, with elevated plasma levels of IL-6, IL-1, and TNF-α, as well as ferritin and other inflammatory biomarkers, has been considered as a typical sign of sHLH/MAS, but the other “key feature” of COVID-19—the progressive lymphopenia with T cell exhaustion [4–6]—has largely been neglected. Of note, both CD4+ and CD8+ T lymphocytes were found to be remarkably decreased in severe cases (median 177.5 and 89.0 × 10^6^/L, respectively), when compared to moderate ones (median 381.5 and 254.0 × 10^6^/L, respectively), thus suggesting T cell lymphopenia may constitute a potential prognostic marker to be included in the monitoring of COVID-19 patients [4]. Frequencies of IFN-γ-producing CD4+ T cells (i.e., cytotoxic Th1 subset) tended to be lower in severe than in moderate illness (median 14.1% versus 22.8%, respectively), possibly indicating a progressive skew of the Th1/Th2 balance toward a tolerogenic response [4]. In addition, the percentages of both memory Th cells and regulatory T cells were found to decrease in severe cases [5].

Nonetheless, in patients with severe systemic hyper-inflammatory diseases driven by other viral infections, hemophagocytic syndrome can be expected as a rare but life-threatening event, and, indeed, sHLH has been recognized to occur in up to 4.3% of sepsis cases [1]. Hence, in those COVID-19 patients showing massive hyperinflammation, a clinical diagnosis of sHLH/MAS may be appropriate and deserves further investigation at the histological level.

More recently, COVID-19 clinical syndrome and related immunopathogenesis have been compared with sepsis, recalling the need to target the underlying and shared impairment of protective T cell immunity, while suppressing the emergent cytokine storm [7–9]. In fact, severe COVID-19 has appeared as a peculiar clinicopathologic entity—yet poorly understood from a mechanistic viewpoint—which however, by definition, may represent a novel form of viral sepsis, being characterized by (a) *T cell deficiencies*, with early and progressive lymphopenia; (b) *systemic hyperinflammation*, with a peculiar time-course, often increasing at a late phase, when coagulopathy and fatal organ damage may eventually occur; and (c) *COVID-19-associated coagulopathy*, displaying some unique clinical and laboratory findings, compared with either disseminated intravascular coagulation or sepsis-induced coagulopathy [10]. Further investigations are required to shed light on the relationships between these clinic-immunologic features and organ failure, possibly paving the way to the treatment (or even prevention) of severe COVID-19, by modulation of host immune system with targeted immunotherapeutic drugs.

During the last few years, cancer immunotherapy with immune checkpoint inhibitors (ICIs), such as anti-PD1/PD-L1 and anti-CTLA-4 monoclonal antibodies (e.g., nivolumab and ipilimumab, respectively), has allowed impressive restoration of T cell immunity against neoplastic cells, which commonly induce overexpression of PD-1/CTLA-4 ligands to foster T cell exhaustion/anergy and break anti-tumor immune surveillance. Intriguingly, several human viruses have been demonstrated to adopt such “cancer-like” immune-evasion strategies, mainly by upregulation of PD-L1 in infected cells, in order to hamper antiviral T cell responses and make a productive infection [11]. Recently, in the attempt to improve antiviral T cell immunity in COVID-19 patients, clinical trials have started to test such T cell activating treatments. Of note, an ongoing Spanish phase 2 study (NCT04335305) seems the first to evaluate the attractive strategy of combining anti-cytokine treatments with ICIs (namely, tocilizumab plus pembrolizumab). Alongside monoclonal antibodies activating T lymphocytes, it has also been suggested that the infusion of SARS-CoV-2-specific cytotoxic T lymphocytes, deriving from HLA-matched convalescent donors, could be explored as innovative cell therapy for COVID-19 [12]. Actually, to maximize potential benefits of different immunotherapeutic approaches against COVID-19, adequate patients’ selection is warranted, possibly performed on the basis of putative biomarkers and immune profiles predictive of response. In addition, by considering the typical disease course, often prolonged for several weeks, the optimal timing for these treatments should be defined.

Thus, it seems conceivable that, during SARS-CoV-2 infection, especially in elderly patients and less frequently in young people, something can go wrong at the delicate interface between effective viral clearance and T cell tolerance. Indeed, COVID-19 may be characterized by different clinical pictures, ranging from almost asymptomatic/mild infections in children and young individuals to lethal “sepsis-like” illness with SARS, particularly in advanced age. What differs between these two distinct stages of life, with regard to the antiviral response toward SARS-CoV-2 infection? Generally, in young subjects and even more in children, T cell immunity is known to be more pronounced and active, especially in terms of lymphocyte counts and adequate antiviral responses, while aged individuals typically undergo a well-described decline in T cell functions, which correlates with higher susceptibility to life-threatening infections, autoimmunity, and cancer [13]. Susceptibility to SARS-CoV-2 infection, related to the different functions and proportions of CD27^dull^ and CD27^bright^ memory B cells, throughout life, has also recently been suggested [14].

**Fig. 1:**
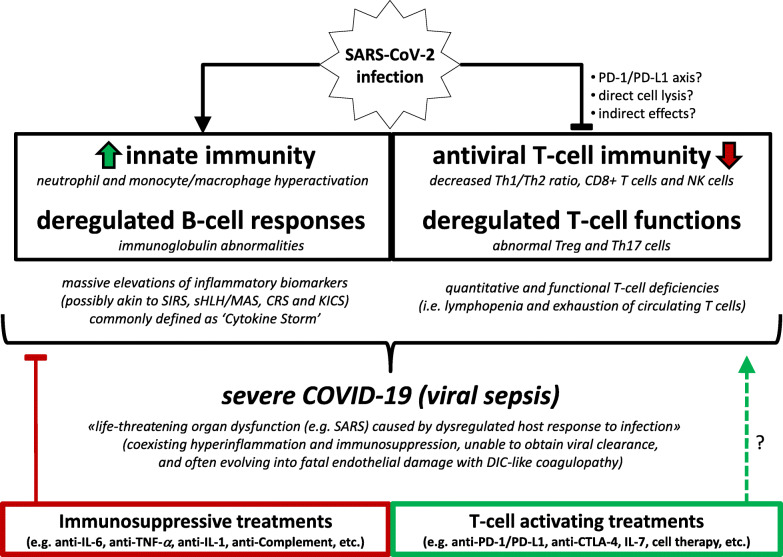
Working model for COVID-19 immunopathogenesis and related immunomodulatory treatments. Acronyms: SARS-CoV-2 severe acute respiratory syndrome-coronavirus-2, SARS severe acute respiratory syndrome, SIRS severe inflammatory response syndrome, sHLH secondary hemophagocytic lymphohistiocytosis, MAS macrophage activation syndrome, CRS cytokine release syndrome, KICS Kaposi sarcoma herpesvirus-associated inflammatory cytokine syndrome, COVID-19 coronavirus disease-19, DIC disseminated intravascular coagulation

In the fight against SARS-CoV-2 pandemic, a more comprehensive vision of COVID-19 immunopathogenesis and related clinical manifestations is warranted to reconcile COVID-19 hyper-inflammatory features—similarly observed in sepsis, sHLH/MAS, and cytokine release syndrome (CRS) [3] induced by chimeric antigen receptor (CAR) T cell therapy, as well as in Kaposi sarcoma herpesvirus-associated inflammatory cytokine syndrome (KICS) [15] occurring in immunocompromised patients—with a renewed pivotal role played by the impairment of antiviral T cell functions. In this perspective (Fig. 1), in parallel with targeted immunosuppressive strategies, an effective reversal of T cell impairment by immune-activating treatments should allow to improve viral clearance and promote a better disease control with faster resolution, probably more akin to what naturally occurs in children infected with SARS-CoV-2.

## Conclusions

SARS-COV-2 has arisen as a new pathogen frequently inducing sepsis-like manifestations in the host. Indeed, based on actual evidence showing hyperinflammation as well as T cell deficiencies and coagulation abnormalities, associated with life-threatening organ dysfunction, severe COVID-19 may be well consistent with a clinical diagnosis of viral sepsis, rather than with a mere hyper-inflammatory disease. This conceptual framing may help to improve clinical management of severe COVID-19 patients, by providing a rationale for the development of novel balanced immunomodulatory approaches, combining both suppressive and activating immunotherapies.

## Data Availability

Not applicable.
